# Supplementary material for: Structural basis for two metal-ion catalysis of DNA cleavage by Cas12i2
Source: Nat Commun. 2020 Oct 16;11:5241. doi: 10.1038/s41467-020-19072-6 (PMC7567891; doi:10.1038/s41467-020-19072-6)
Supplement: Supplementary file 1 — Supplementary Information [file 41467_2020_19072_MOESM1_ESM.pdf]

SUPPLEMENTARY INFORMATION

Structural basis for two metal-ion catalysis of DNA Cleavage by Cas12i2

Xue Huang et al.

TABLE OF CONTENTS

1. Supplementary Tables

Supplementary table 1. DNA and RNA Sequences Used in This Study

2. Supplementary Figures

Supplementary Figure 1. Crystal structure of Cas12i2-crRNA-DNA ternary complex.

Supplementary Figure 2. The crRNA repeat recognition.

Supplementary Figure 3. Interactions between Cas12i2 and nucleic acids.

Supplementary Figure 4. The ssDNA and Mg^{2+} ion bound in the RuvC catalytic pocket.

Supplementary Figure 5. Structural comparison of Cas12i2 complexes in distinct states.

Supplementary Figure 6. DNA cleavage assay in the presence of ssDNA activator.

Supplementary Figure 7. Structural comparison of Cas12i2 and Cas12b.

Supplementary table 1. DNA and RNA Sequences Used in This Study

IDENTIFIER	Sequence (5' to 3')	SOURCE
Cas12i2-crRNA-dsDNA (26 nt)	TS: AATAATGTCACCCTGCTTGCTCTGTTGAAAGCGGC	Sangon Biotech
	NTS: GCCGCTTTCTT	Sangon Biotech
Cas12i2^{E833A}-crRNA-dsDNA (12 nt)	TS: GCTTGCTCTGTTGAAAGCGGC	Sangon Biotech
	NTS: GCCGCTTTCTT	Sangon Biotech
dsDNA-31 nt PAM TTC sequence cloned in pUC19 in Figures 2d, 2e, 2f, 3d, 3e, 3f, 4c, 4e and Supplementary figure 2c, 6a, 6f	TS:GATTAAATAATGTCACCCTGCTTGCTCTGTTGAAAGCGGC	Sangon Biotech
	NTS:GCCGCTTTCAACAGAGCAAGCAGGGTGACATTATTTAATC	Sangon Biotech
dsDNA-PAM ATC sequence cloned in pUC19 in Figure 3e	TS:GATTAAATAATGTCACCCTGCTTGCTCTGTTGATAGCGGC	Sangon Biotech
	NTS:GCCGCTATCAACAGAGCAAGCAGGGTGACATTATTTAATC	Sangon Biotech
dsDNA-PAM GTC sequence cloned in pUC19 in Figure 3e	TS:GATTAAATAATGTCACCCTGCTTGCTCTGTTGACAGCGGC	Sangon Biotech
	NTS:GCCGCTGTCAACAGAGCAAGCAGGGTGACATTATTTAATC	Sangon Biotech
dsDNA-PAM CTC sequence cloned in pUC19 in Figure 3e	TS:GATTAAATAATGTCACCCTGCTTGCTCTGTTGAGAGCGGC	Sangon Biotech
	NTS:GCCGCTCTCAACAGAGCAAGCAGGGTGACATTATTTAATC	Sangon Biotech
dsDNA-PAM TAC sequence cloned in pUC19 in Figure 3e	TS:GATTAAATAATGTCACCCTGCTTGCTCTGTTGTAAGCGGC	Sangon Biotech
	NTS:GCCGCTTACAACAGAGCAAGCAGGGTGACATTATTTAATC	Sangon Biotech
dsDNA-PAM TGC sequence cloned in pUC19 in Figure 3e	TS:GATTAAATAATGTCACCCTGCTTGCTCTGTTGCAAGCGGC	Sangon Biotech
	NTS:GCCGCTTGCAACAGAGCAAGCAGGGTGACATTATTTAATC	Sangon Biotech
dsDNA-PAM TCC sequence cloned in pUC19 in Figure 3e	TS:GATTAAATAATGTCACCCTGCTTGCTCTGTTGGAAGCGGC	Sangon Biotech
	NTS:GCCGCTTCCAACAGAGCAAGCAGGGTGACATTATTTAATC	Sangon Biotech
dsDNA-PAM ATTC sequence cloned in pUC19 in 3f	TS:GATTAAATAATGTCACCCTGCTTGCTCTGTTGAATGCGGC	Sangon Biotech
	NTS:GCCGCATTCAACAGAGCAAGCAGGGTGACATTATTTAATC	Sangon Biotech
dsDNA-PAM GTTC sequence cloned in pUC19 in 3f	TS:GATTAAATAATGTCACCCTGCTTGCTCTGTTGAACGCGGC	Sangon Biotech
	NTS:GCCGCGTTCAACAGAGCAAGCAGGGTGACATTATTT	Sangon Biotech

	AATC	
dsDNA-PAM CTTC sequence cloned in pUC19 in 3f	TS:GATTAAATAATGTCACCCTGCTTGCTCTGTTGAAGGCGGC	Sangon Biotech
	NTS:GCCGCCTTCAACAGAGCAAGCAGGGTGACATTATTTAATC	Sangon Biotech
5’ Cy3- labeled nonspecific cleavage ssDNA substrate in Figures 6a, 6b, 6c, Supplementary Figures 6b and 6c	Cy3-CTCAGTGATCTAAAATCATATGTAAAGTTAAATAGCAGAGTGACCTGTCATGA	Sangon Biotech
ssDNA1 in Figure 6a	NTS:GCCGCTTTCAACAGAGCAAGCAGGGTGACATTATTTAATC	Sangon Biotech
ssDNA2 in Figure 6a	TS:GATTAAATAATGTCACCCTGCTTGCTCTGTTCTTAGCGGC	Sangon Biotech
ssDNA3 in Figure 6a	TS:GATTAAATAATGTCACCCTGCTTGCTCTGTTGAAAGCGGC	Sangon Biotech
ssDNA4 in Figure 6a	TS: GATTAAATAATGTCACCCTGCTTGCTCTGTT	Sangon Biotech
dsDNA1 in Figure 6a	TS:GATTAAATAATGTCACCCTGCTTGCTCTGTTGAAAGCGGC	Sangon Biotech
	NTS:GCCGCTTTCAACAGAGCAAGCAGGGTGACATTATTTAATC	Sangon Biotech
dsDNA2 in Figure 6a	TS:GATTAAATAATGTCACCCTGCTTGCTCTGTTGAAAGCGGC	Sangon Biotech
	NTS:GCCGCTTTCTTGTCTCGTTCGTCCCACTGATTATTTAATC	Sangon Biotech
dsDNA3 in Figure 6a	TS:GATTAAATAATGTCACCCTGCTTGCTCTGTTCTTAGCGGC	Sangon Biotech
	NTS:GCCGCTAAGTTGTCTCGTTCGTCCCACTGATTATTTAATC	Sangon Biotech
TS 31 nt in Figure 6b, 6c	TS: GATTAAATAATGTCACCCTGCTTGCTCTGTT	Sangon Biotech
TS 20 nt in Figure 6c	TS: GTCACCCTGCTTGCTCTGTT	Sangon Biotech
TS M 1-5 nt in Figure 6c	TS: GTCACCCTGCTTGCTGACAA	Sangon Biotech
TS M 6-10 nt in Figure 6c	TS: GTCACCCTGCAACGACTGTT	Sangon Biotech
TS M 11-15 nt in Figure 6c	TS: GTCACGGACGTTGCTCTGTT	Sangon Biotech
TS M 16-20 nt in Figure 6c	TS: CAGTGCCTGCTTGCTCTGTT	Sangon Biotech
pre-crRNA in Figures 6d, 6e and Supplementary Figure 6d	GGUAUAUAGAAAUCCGUCUUUCAUUGACGGAACAGAGCAAGCAGGGUGACAUUAUUUAAUC	N/A
pre-crRNA (A-25U) in Figure 6d	GGUAUUUAGAAAUCCGUCUUUCAUUGACGGAACAGAGCAAGCAGGGUGACAUUAUUUAAUC	N/A
pre-crRNA (U-24G) in Figure 6d	GGUAUAGAGAAAUCCGUCUUUCAUUGACGGAACAGAGCAAGCAGGGUGACAUUAUUUAAUC	N/A
pre-crRNA (A-23U) in Figure 6d	GGUAUAUUGAAAUCCGUCUUUCAUUGACGGAACAGAG	N/A

	CAAGCAGGGUGACAUUAUUUAAUC	
pre-crRNA (G-22U) in Figure 6d	GGUAUAUAUAAAUCCGUCUUUCAUUGACGGAACAGAG CAAGCAGGGUGACAUUAUUUAAUC	N/A
pre-crRNA (A-21U) in Figure 6d	GGUAUAUAGUAAUCCGUCUUUCAUUGACGGAACAGAG CAAGCAGGGUGACAUUAUUUAAUC	N/A
pre-crRNA (A-20U) in Figure 6d	GGUAUAUAGAUAUCCGUCUUUCAUUGACGGAACAGAG CAAGCAGGGUGACAUUAUUUAAUC	N/A
crRNA	GGAGAAAUCCGUCUUUCAUUGACGGAACAGAGCAAGC AGGGUGACAUUAUUUAAUC	N/A
RNA activator sequence in Supplementary Figure 6b	GGAUUAAAUAAUGUCACCCUGCUUGCUCUGUUC	N/A
DNA activator sequence in Supplementary Figure 6b	GTCACCCTGCTTGCTCTGTT	Sangon Biotech

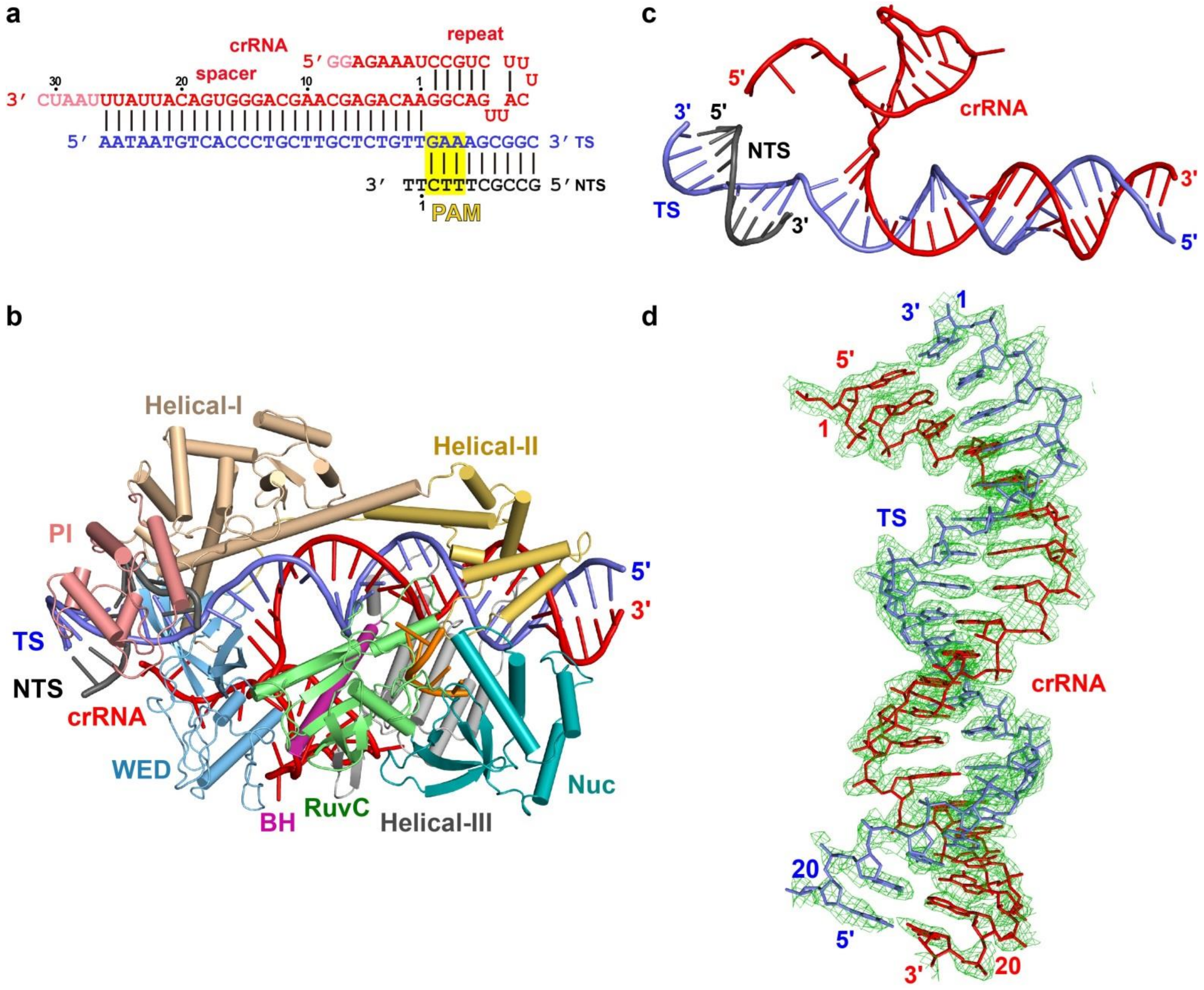

Supplementary Figure 1. Crystal structure of Cas12i2-crRNA-DNA ternary complex.

a. The guide RNA and target DNA nucleotide sequences used for Cas12i2 co-crystallization. The ordered and disordered crRNA nucleotides are shown in red and light red, respectively. The target strand DNA and non-target strand DNA are shown in blue and black.

b. The crystal structure of the Cas12i2^{E833A}-crRNA-26 nt dsDNA ternary complex (PDB ID: 6LTR).

c. Structure of crRNA and DNA in the Cas12i2^{E833A}-crRNA-DNA ternary complex.

d. The 2Fo-Fc electron density map of the partial crRNA:DNA heteroduplex (in sticks) is shown as a green mesh (contoured at 1.5 σ).

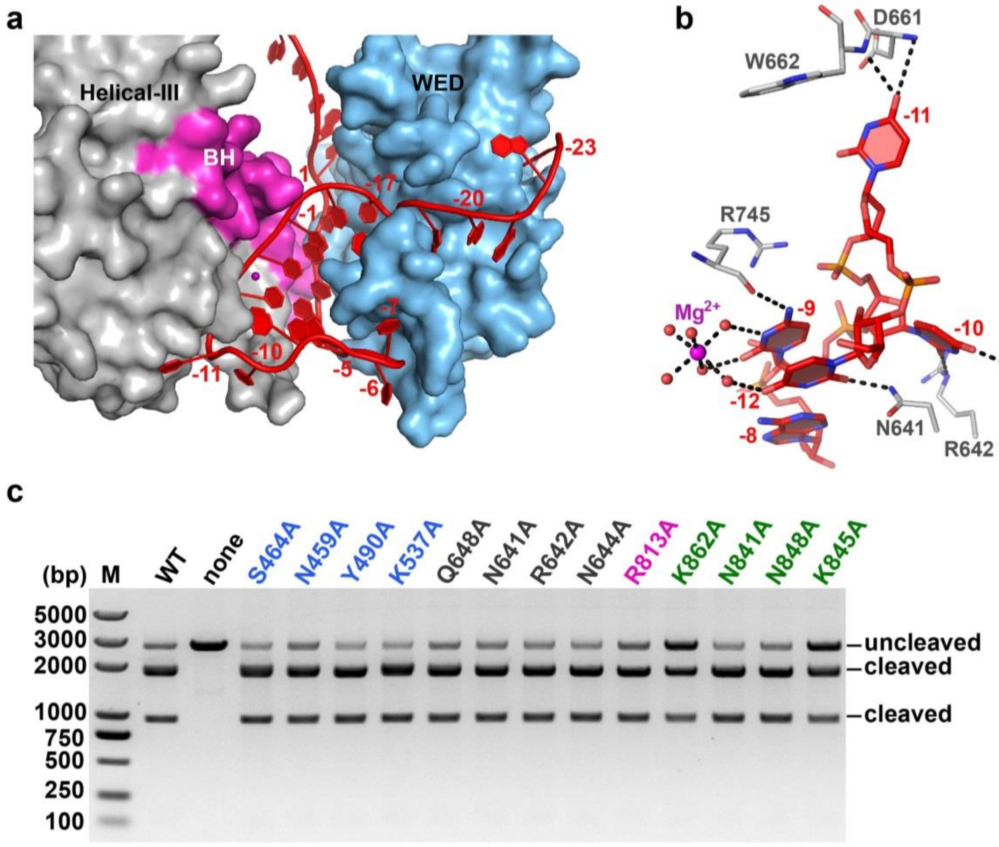

Supplementary Figure 2. The crRNA repeat recognition.

a. The repeat region of the crRNA is positioned in cleft between the Helical-III and WED domains.

b. The closed up view of the interactions between Cas12i2 and the loop within the repeat.

c. Mutational analysis of amino acids interacting with the crRNA using the linear plasmid DNA. 200 nM wild-type and mutated Cas12i2 were incubated with crRNA at a molar ratio of 1:1.1 on ice for 30 min to allow binary complex formation, followed by the additional of 300 ng pre-linearized plasmid DNA substrate. The final reaction was incubated for 15 min at 37°C. Cleavage products were analyzed by agarose gel electrophoresis.

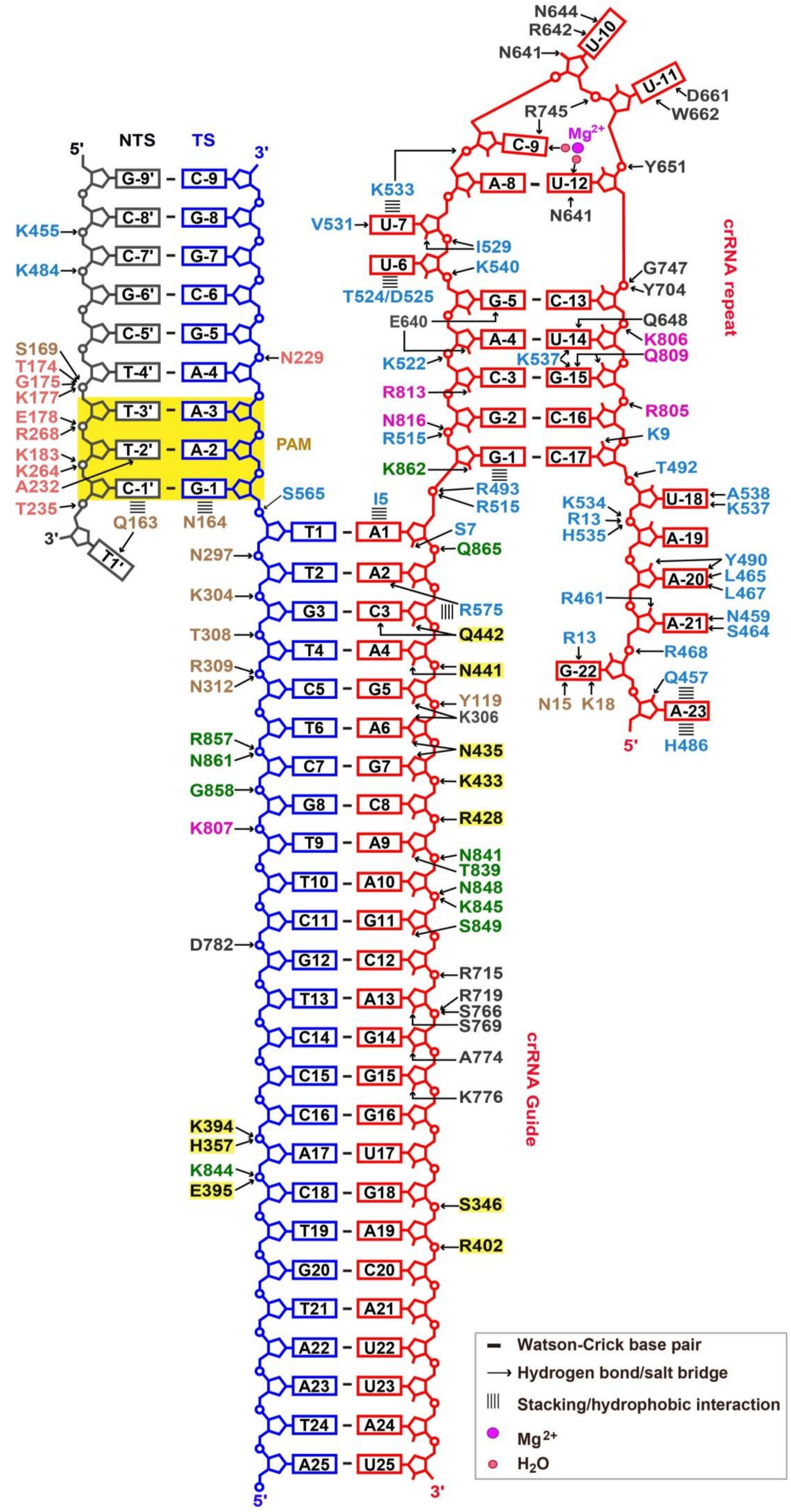
NTS
TS
5'
3'
G-9'
C-9
C-8'
G-8
C-7'
G-7
G-6'
C-6
C-5'
G-5
T-4'
A-4
T-3'
A-3
T-2'
A-2
C-1'
G-1
T1'
PAM
K455
K484
S169
T174
G175
K177
E178
R268
K183
K264
A232
T235
Q163
N164
N229
S565
T1
T2
G3
T4
C5
T6
C7
G8
T9
T10
C11
G12
T13
C14
C15
C16
A17
C18
T19
G20
T21
A22
A23
T24
A25
A1
A2
C3
A4
G5
A6
G7
C8
A9
A10
G11
C12
A13
G14
G15
G16
U17
G18
A19
C20
A21
U22
U23
A24
U25
N297
K304
T308
R309
N312
R857
N861
G858
K807
D782
K394
H357
K844
E395
I5
S7
Q865
R575
Q442
N441
Y119
K306
N435
K433
R428
N841
T839
N848
K845
S849
R715
R719
S766
S769
A774
K776
S346
R402
crRNA Guide
N644
R642
N641
U-10
U-11
D661
W662
R745
Mg2+
C-9
A-8
U-12
Y651
K533
V531
U-7
I529
U-6
K540
T524/D525
G-5
C-13
G747
Y704
E640
Q648
A-4
U-14
K806
K522
K537
Q809
C-3
G-15
R813
G-2
C-16
R805
N816
R515
K9
K862
G-1
C-17
T492
R493
R515
U-18
A538
K537
K534
R13
H535
A-19
Y490
L465
A-20
L467
R461
A-21
N459
S464
R13
R468
G-22
Q457
N15
K18
A-23
H486
crRNA repeat
Watson-Crick base pair
Hydrogen bond/salt bridge
Stacking/hydrophobic interaction
Mg2+
H2O

Supplementary Figure 3. Interactions between Cas12i2 and nucleic acids.

Schematic interactions between Cas12i2, crRNA, and DNA. The domains of specific Cas12i2 residues are color-coded as indicated.

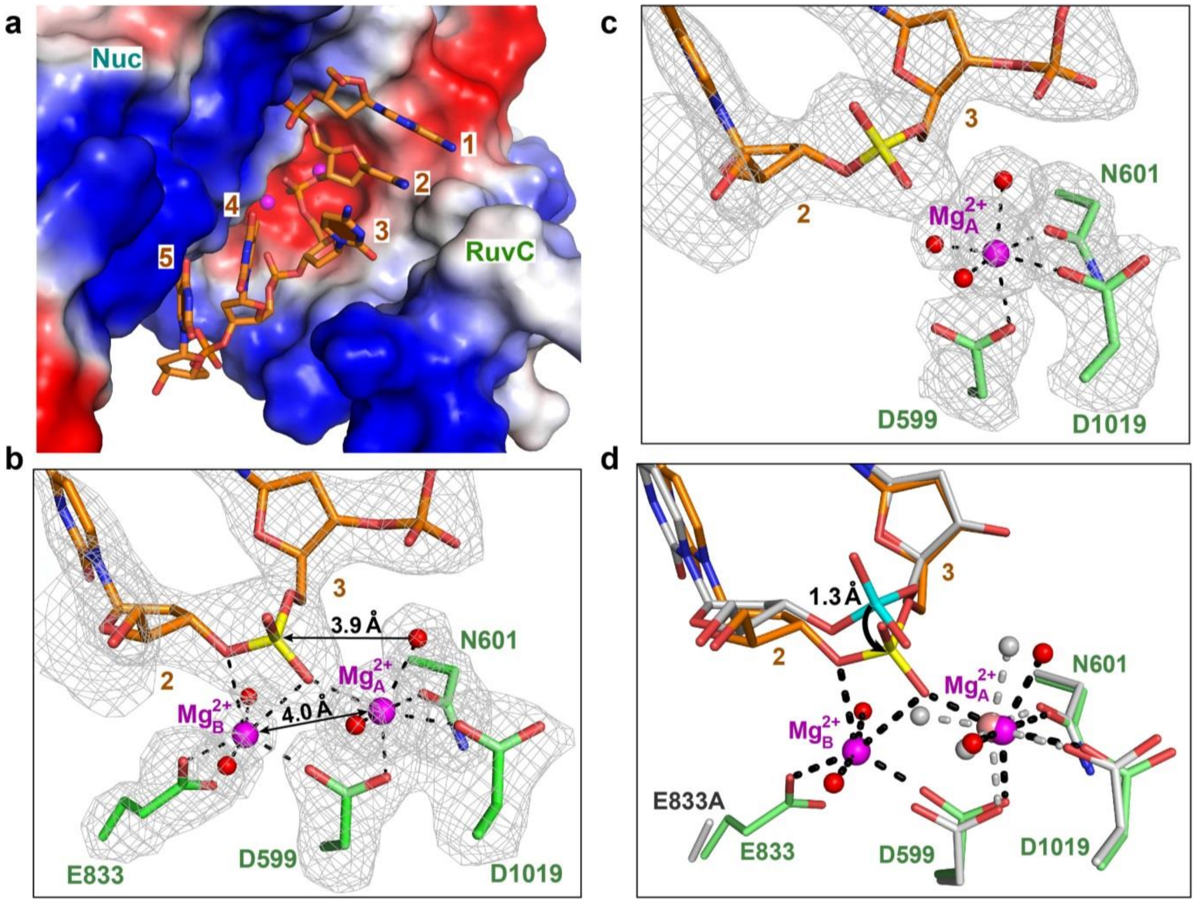

Supplementary Figure 4. The ssDNA and Mg^{2+} ion bound in the RuvC catalytic pocket.

a. The 5-mer ssDNA (in orange) is positioned in a positively charged cleft formed by the RuvC and Nuc domains.

b. Two Mg^{2+} ions bound in the catalytic pocket of the Cas12i2^{wt}-crRNA-dsDNA ternary complex. The Fo-Fc omit electron density map of two Mg^{2+} ions, DNA substrate, water molecules and amino acids coordinating with Mg^{2+} ions is shown as a gray mesh (contoured at 4.0 σ).

c. One Mg^{2+} ions bound in the Cas12i2^{E833A}-crRNA-dsDNA ternary complex. The Fo-Fc omit electron density map of a Mg^{2+} ion, DNA substrate, water molecules and amino acids is shown in gray mesh (contoured at 4.0 σ).

d. The structural comparison between the wild type and Cas12i2^{E833A} ternary complexes, showing that the substrate DNA undergoes conformational changes upon binding of two divalent metals. The catalytic residues, Mg^{2+}, scissile phosphate and DNA within the Cas12i2^{E833A} ternary complex are in grey, pink, cyan and grey respectively. The catalytic residues, Mg^{2+}, scissile phosphate and DNA within the Cas12i2^{wt} ternary complex are colored coded as panel b.

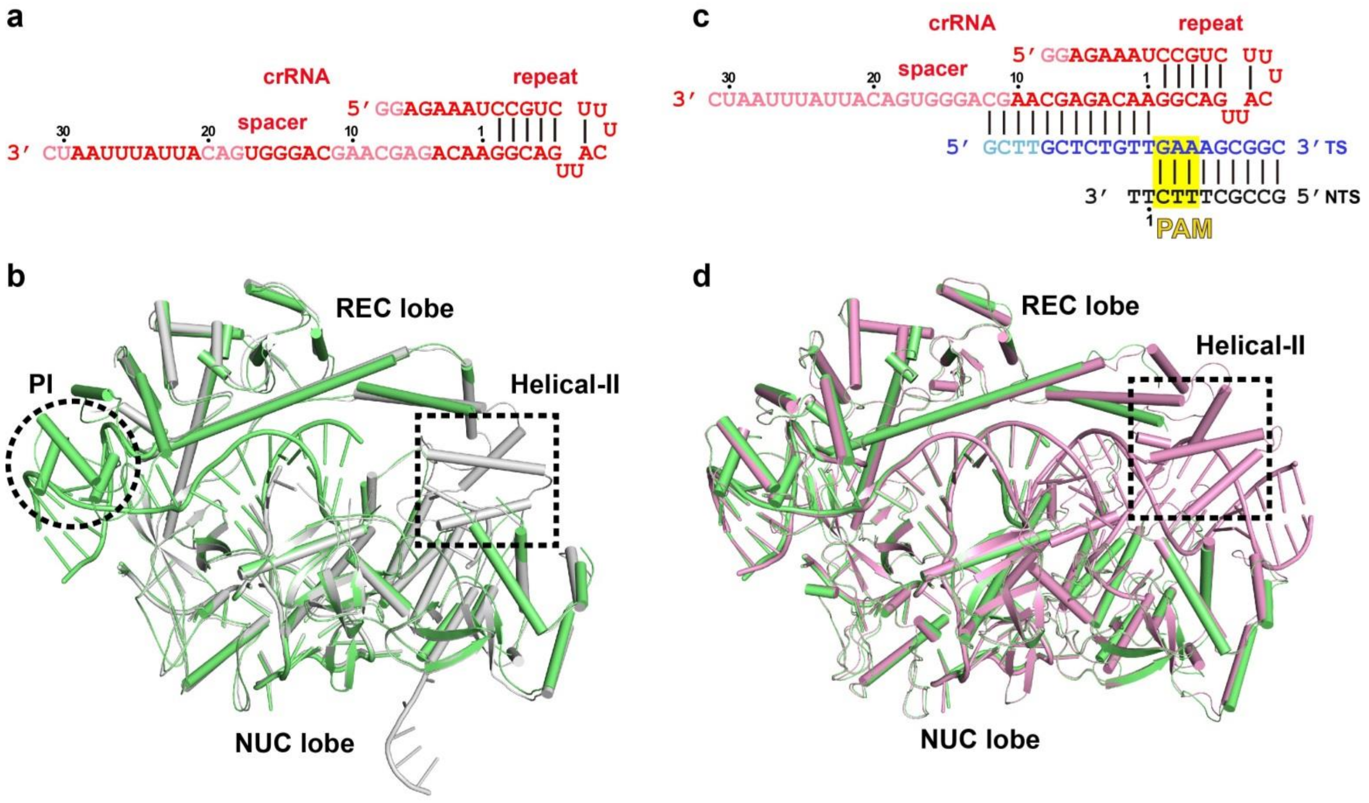

Supplementary Figure 5. Structural comparison of Cas12i2 complexes in distinct states.

a. The sequence of guide RNA used for Cas12i2 co-crystallization. The disordered nucleotides are shown in light red.

b. Superposition of Cas12i2 in the binary complex (gray) and in the 12-nt DNA bound ternary complex (green).

c. The guide RNA and target DNA nucleotide sequences used for Cas12i2 co-crystallization. The disordered nucleotides of the crRNA and target strand are shown light red and light blue, respectively.

d. Superposition of Cas12i2 in the partial-paired (green) and the fully-paired (pink) DNA-bound ternary complexes.

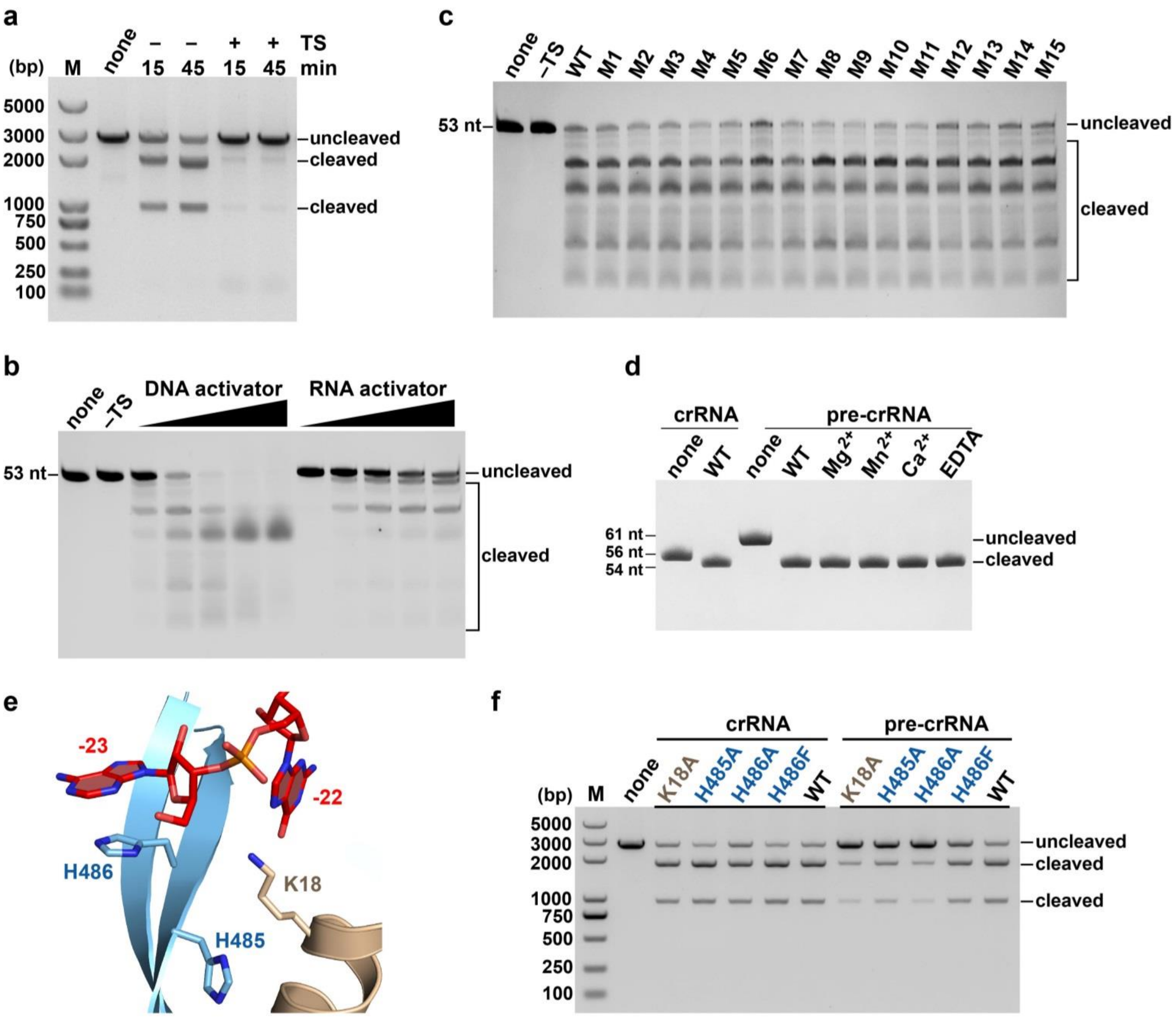

Supplementary Figure 6. DNA cleavage assay in the presence of ssDNA activator.

a. DNA cleavage assay using the linear plasmid DNA, showing the preformed Cas12i2-crRNA-ssDNA complex cannot cleave dsDNA.

b. The DNA cleavage assay using the 5'-Cy3 labeled ssDNA in the presence of ssDNA target or ssRNA activator.

c. A single nucleotide mismatched within the ssDNA activator has no effect on the ssDNA cleavage in *trans*.

d. Denaturing gel analysis showing that Cas12i2 cleaves pre-crRNA in a metal-independent manner. 20 μM Cas12i2 was incubated with 20 μM pre-crRNA substrate, and divalent cations as indicated or EDTA at 37°C for 30 min.

e. Magnified view of the catalytic center of the crRNA processing.

f. DNA cleavage assay showing that the mutation of pre-crRNA processing amino acids reduces the DNA cleavage.

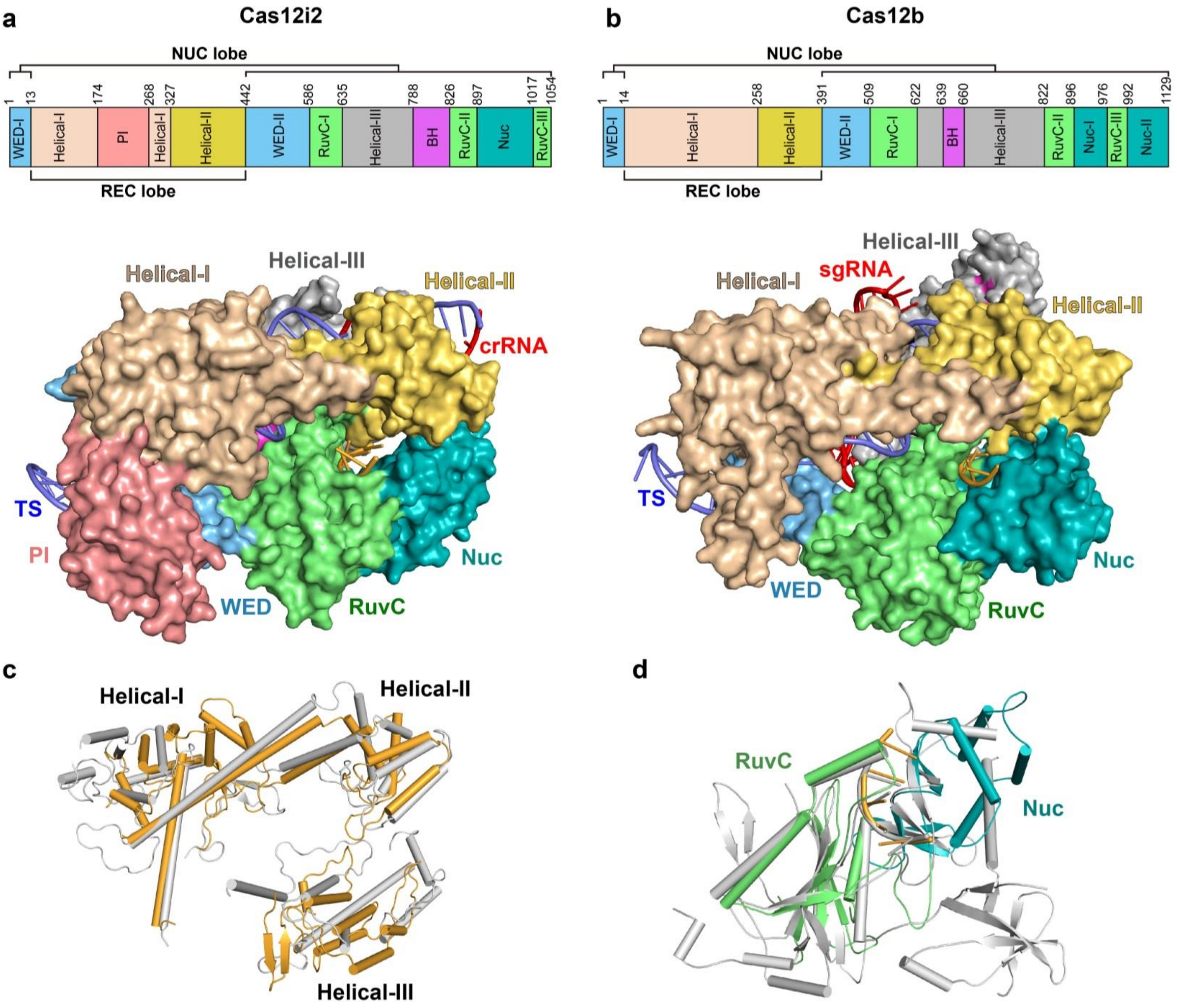

Supplementary Figure 7. Structural comparison of Cas12i2 and Cas12b.

a. The Cas12i2 domain organization and the crystal structure of the Cas12i2-crRNA-DNA ternary complex.
b. The Cas12b domain organization and the structure of the AacCas12b-sgRNA-DNA ternary complex.
c. Superposition of REC lobe of Cas12i2 (in orange) and AacCas12b (in gray).
d. Superposition of the RuvC and NuC domains of Cas12i2 (in green and cyan) and AacCas12b (in gray).
